# Multiple sclerosis burden and quality of care index in China and the G20 (1990–2021): A cross-regional analysis using global burden of disease database 2021

**DOI:** 10.1097/MD.0000000000047260

**Published:** 2026-01-23

**Authors:** Haosen Liao, Xiaomin Zhu, Cuilan Chen, Linglu Dun, Yingrui Huang, Yuehui Ma, Honghu Wang, Hongen Yan, Guanhua Li, Zheyi Zhou

**Affiliations:** a Liuzhou Hospital of Traditional Chinese Medicine Affiliated to the Guangxi University of Chinese Medicine, Liuzhou, Guangxi, China; b Graduate School of Guangxi University of Chinese Medicine, Nanning, China; c The First Clinical College of Medicine, Guangxi University of Chinese Medicine, Nanning, China.

**Keywords:** epidemic study, global burden of disease, multiple sclerosis, quality of care index

## Abstract

Multiple sclerosis (MS) is a chronic immune-mediated disease of the central nervous system with a growing global burden. While traditionally considered rare in Asia, China rising MS prevalence and disproportionate disability present major public health challenges. Comprehensive cross-regional comparisons between China, the group of 20 (G20), and global aggregates remain limited. Using data from the global burden of disease 2021 study, we quantified MS incidence, prevalence, mortality, and disability-adjusted life years (DALYs) in China, G20 countries, and worldwide from 1990 to 2021. Temporal trends were analyzed with joinpoint regression, drivers of burden were decomposed into epidemiological change, population aging, and population growth, and future trajectories were forecast to 2050 using autoregressive integrated moving average (ARIMA) models. To evaluate healthcare system performance, we further constructed a Quality of Care Index (QCI) based on composite ratios of incidence, prevalence, mortality, and DALYs. From 1990 to 2021, MS prevalence and DALYs increased substantially in all regions, with China exhibiting faster growth in DALYs than G20 or global averages, particularly among women and working-age adults. In contrast, the G20 showed a more pronounced aging of MS burden. Decomposition analysis revealed that population aging and epidemiological change were the primary drivers of China rising burden. Forecasting predicts continued growth of cases and DALYs through 2050. QCI analysis demonstrated global improvement in MS care quality, with China achieving marked progress, especially among elderly groups; however, persistent disparities remain, with lower QCI values in children, older adults, and women, showing a consistent “middle-high, both-ends-low” pattern across regions. The MS burden in China has expanded more rapidly than in the G20 or globally, with disproportionate impacts on women and working-age populations. Although care quality has improved, inequities by age and sex persist. These findings highlight the urgent need for earlier diagnosis, equitable access to disease-modifying treatments, and targeted interventions for vulnerable groups to mitigate the future burden of MS.

**Highlights**

1. China MS burden has increased rapidly (EAPC: prevalence + 1.8%, DALYs + 1.7%), while global and G20 rates declined.
2. A significant reversal in the previously declining global trend of MS DALYs was observed beginning in 2019.
3. China MS burden is concentrated in younger adults (68.3% of DALYs in ages 15–49), contrasting with aging-driven patterns in G20 countries.
4. A 2-step methodology was used to construct the quality of care index (QCI), incorporating principal component analysis (PCA) to extract and scale indicators.
5. Projections indicate continued growth of MS prevalence in China through 2050, particularly among women.

## 1. Introduction

Multiple sclerosis (MS) is a chronic, immune‑mediated inflammatory disease of the central nervous system, exhibits a progressively escalating global disease burden. According to the third edition of the Atlas of MS, the global prevalence rose from 29.26 to 43.95 per 100,000 people between 2013 and 2020, representing a 14.69% increase, with marked variation by latitude.^[1–3]^ Notably, the geographical distribution of MS displays pronounced differences, higher prevalence is generally observed in high‑income countries compared with low‑ and middle‑income regions. Although Asia has historically been considered a low‑risk region for MS, the absolute number of cases in China – given its large population and geographic diversity – poses a growing challenge to public health systems.^[2]^

The burden of MS varies substantially between regions. In China, the age‑standardized prevalence rate (ASPR) increased by 18.7% from 1990 to 2019, a slower rate than that seen in high‑income countries in Europe and North America. However, China disability‑adjusted life years (DALYs) growth rate ranks among the highest globally, suggesting a mismatch between disease severity and healthcare resources.^[2,4]^ Similar disparities are observed in other developed settings. For example, prevalence increased by 31% in Australia over 10 years, reaching 124.8 per 100,000; by 40% in northern Japan over 2 decades; and by 42% in Italy between 2011 and 2015. On average, prevalence in developed countries is approximately 5 times that in developing countries.^[5–8]^ Of greater concern are trends in specific populations: the prevalence of MS is significantly higher in women of reproductive age (15–49 years) than in men (about 3:1). Common comorbidities – including depression, anxiety, migraine, and reproductive health disorders – can worsen disability progression, recurrence risk, quality of life, healthcare needs, and mortality.^[9,10]^ In developed countries, MS prevalence among children has risen to 3% to 10%, often accompanied by polypharmacy; around 67% of pediatric patients take 5 or more medications, increasing the risk of drug–drug interactions.^[11–13]^

There is a pressing need to understand the drivers of MS‑related disability. In Japan, which shares similar latitude with northern China, a 2024 national survey reported a disability rate of 62.3% among patients.^[14]^ This process may be modulated by multidimensional factors. First, the core symptom cluster directly reduces quality of life: 80% of patients suffer from fatigue, the primary disabling factor.^[15]^ Other factors include gender-differentiated insomnia (self-reported rate of 52%; 95% confidence interval (CI): 44%–59%; women >men), dysphagia triggering aspiration pneumonia (prevalence of 41%; 95% CI: 34%–49%), and highly heterogeneous dementia (comorbidity of 5.31%, 95% CI: 2.25%–11.98%).^[16–18]^ Second, accelerated disability occurs through disease transition. Approximately 50% of patients progress to secondary progressive MS (SPMS) within 15 years of onset. The risk of transformation is significantly influenced by external factors, such as behavioral patterns and geography.^[19–22]^ Ultimately, Genetic susceptibility, environmental exposures, and diagnostic disparities likely interact to accelerate disability.^[23,24]^

Early initiation of disease‑modifying treatments (DMTs) can reduce neurological damage and improve long‑term outcomes. However, high rates of misdiagnosis (5%–41%) and underdiagnosis (3%–58%) delay treatment initiation.^[23,25]^ Particularly concerning are diagnostic delays in China, where patients experience prolonged intervals ranging from 5 to 10 years (median delay: 34.6 months), and women are more than twice as likely as men to be misdiagnosed.^[23,26,27]^ Differentiating MS from neuromyelitis optical spectrum disorders (NMOSD) is particularly important in Asian populations.^[14]^ Furthermore, current DMTs have limited efficacy for progressive MS, underscoring the need for earlier diagnosis and improved disease management.^[28–30]^

The group of 20 (G20) integrates 19 sovereign states and the European Union – collectively representing major economies including China, G7 nations, and key emerging markets (Argentina, Brazil, India, Indonesia, Mexico, Russia, South Korea).^[31]^ Critically, this coalition accounts for 85 percent of global GDP and encompasses approximately 65 percent of both the world’s population and terrestrial territory, thereby constituting a geopolitically influential, geographically comprehensive, and socioeconomically heterogeneous cohort. Given its unique integration of developed and developing economies, systematic comparisons of disease burden trajectories between China, the G20 collective, and global aggregates offer substantive insights into health-economy nexuses.^[31]^ Yet, despite this analytical imperative, extant literature lacks comprehensive cross-regional assessments of MS epidemiology across these stratified geopolitical entities. This study aims to investigate the temporal and demographic patterns of MS burden within these populations, identify key drivers, and project future trends to inform targeted prevention and management strategies.

## 2. Methods

### 2.1. Data sources

This study conducted secondary analyses using anonymized, aggregated datasets from the global burden of disease (GBD) 2021 project. The GBD framework quantifies health losses from 371 diseases and injuries across 204 countries and territories, integrating over 100,000 sources, including population‑based registries, disease‑specific registries, epidemiological surveys, healthcare administrative databases, and cohort studies.^[32]^ Therefore, the study population encompasses the entire populations of China, G20 nations, and the global aggregate, without specific participant-level inclusion or exclusion criteria applied by the authors of this paper. The eligibility of source data for inclusion in the GBD models is determined by the Institute for health metrics and evaluation, and detailed metadata for all input sources are available through the global health data exchange (GHDx).

Standardized epidemiological data for MS (ICD‑10 code: G35) were extracted for China, G20 countries, and the global population from 1990 to 2021 via the Global Health Data Exchange (http://ghdx.healthdata.org/). Core indicators included age‑standardized incidence rate (ASIR), prevalence rate (ASPR), mortality rate (ASMR), and disability‑adjusted life years rate (ASDR), each with 95% uncertainty intervals (UIs).

Three analytical approaches were applied: Trend analysis: Joinpoint regression estimated annual percentage change (EAPC) and average APC with 95% CIs.^[2]^ Driver decomposition: A factor decomposition algorithm quantified the contributions of epidemiological changes, population aging, and population size growth to observed changes in incidence, prevalence, DALYs, and mortality.^[3]^ Forecasting: An autoregressive integrated moving average (ARIMA) model was constructed to predict trends to 2050.

All analyses adhered to the reporting of global health estimates (REGAHS) guidelines. ethical approval was not required due to the use of publicly available, de‑identified data.

### 2.2. Statistical analysis

#### 2.2.1. *Long‑term trend estimation*

The EAPC was calculated by fitting a log‑linear regression model to age‑standardized rate (ASR) time‑series data:


$$\begin{array}{l} \text{l}n(ASR)=\beta 0+\beta 1\times \text{Y}ear+\epsilon \end{array}$$

The EAPC and its 95% CI were derived as: EAPC = (*eβ*1 − 1) × 100%. Joinpoint regression (version 4.9.1) identified significant changes in trends, with the optimal number of segments determined by the Bayesian Information Criterion and a Monte Carlo permutation test (α = 0.05). Joinpoint regression was employed specifically to identify the years in which significant changes in the long-term temporal trends of MS burden occurred, without assuming a linear trend throughout the entire study period.

#### 2.2.2. *Factor decomposition of drivers*

ΔR = ΔRepid+ΔRage+ΔRpopΔ*R*=Δ*R*epid+Δ*R*age+Δ*R*pop

Changes in each indicator (ΔR) were decomposed into: epidemiological change (ΔRepid) – shifts in incidence or prevalence independent of demographic change; population aging (ΔRage) – changes due to shifts in age structure; and population growth (ΔRpop) – changes due to absolute population size. This was implemented using a difference‑in‑differences algorithm.

All EAPCs, average APCs, and age-standardized rates are presented alongside their 95% CIs or 95% UIs as applicable.

#### 2.2.3. ARIMA forecasting model

Future trends (2022–2050) were modeled using the Box–Jenkins ARIMA(*p,d,q*) method. The ARIMA model is a widely utilized forecasting tool for time series data, owing to its capacity to identify trends, seasonality, and underlying patterns in disease burden. In earlier studies, ARIMA has been employed with a high degree of success in predicting the incidence and economic burden of chronic diseases, including Alzheimer disease and diet-related stroke.^[33,34]^ This study employs ARIMA to forecast the future burden of MS in China, the G20 and the global context, on the basis of the strong predictive capabilities of the model. Autocorrelation (ACF) and partial autocorrelation (PACF) plots determined the autoregressive (*p*) and moving average (*q*) orders; the augmented Dickey–Fuller test determined differencing order (*d*). Model selection was based on Akaike (AIC) and Bayesian (BIC) information criteria. Residuals were tested for white noise using the Ljung–Box test (*P* >.05).

#### 2.2.4. Uncertainty estimation

GBD 2021 estimates were generated using an ensemble of modeling tools, primarily based on a Bayesian meta-regression framework (DisMod-MR 2.1), which is explicitly designed to address missing data, smooth variation, and extrapolate estimates for locations and years with no primary data. The models borrow strength from spatial and temporal covariates and from data-rich locations to inform estimates for data-sparse regions. Furthermore, as noted in the methods, missing values for the 2020 to 2021 period, which were exacerbated by the pandemic, were interpolated and adjusted using the Institute for Health Metrics and Evaluation algorithm specifically designed to correct for pandemic-related disruptions in health service reporting and utilization.

#### 2.2.5. Quality of care index

The quality of care index (QCI) was developed to assess the quality of care for MS, a metric that has been corroborated in earlier research.^[4]^ Drawing on age-standardized primary measures – including incidence, prevalence, deaths, DALYs, YLLs, and YLDs – from the GBD 2021 study, 4 secondary indices were derived to serve as proxies for evaluating the performance of healthcare systems in managing the burden of MS. These indices were selected as they capture distinct dimensions of care quality:

The formulas used to compute these indices are provided below:


$$\begin{array}{l} prevalence-to-incidence\left(x\right)=\frac{prevalence\left(x\right)}{incidence\left(x\right)} \end{array}$$

This ratio reflects the effectiveness of preventive strategies and long-term disease management, where a higher value denotes better control of the disease.


$$\begin{array}{l} Mortality\;to\;incidence\;ratio\left(MIR\right)\left(x\right)=\frac{Death\left(x\right)}{Incidence\left(x\right)} \end{array}$$

The MIR serves as an indicator of healthcare quality, correlating the number of deaths with new cases. Elevated MIR values are associated with poorer health outcomes and highlight areas requiring clinical improvement.^[35]^


$$\begin{array}{l} DALY-to-prevalence\left(x\right)=\frac{DALY\left(x\right)}{Incidence\left(x\right)} \end{array}$$

This metric evaluates the disease burden per prevalent case. A higher ratio implies a greater health impact on affected individuals.


$$\begin{array}{l} YLL-to-YLD\left(x\right)=\frac{YLL\left(x\right)}{YLD\left(x\right)} \end{array}$$

This ratio contrasts fatal and nonfatal health outcomes. A higher value indicates a greater proportion of premature death relative to years lived with disability. Here, x refers to a specific region or country.

To integrate these 4 indices into a unified measure, we applied principal component analysis (PCA), a multivariate statistical technique for dimensionality reduction.^[36]^ The analysis was performed on the standardized indices. We extracted the first 2 principal components (PC1 and PC2), which together captured the majority of the variance in the data. To form a comprehensive index, a weighted PCA score was calculated for each observation (e.g., country-year-sex group) using the following formula:


$$\begin{array}{l} PCAscore\left(x\right)=\left(\frac{Var\left(PC1\right)}{Var\left(PC1\right)+Var\left(PC2\right)}\right)\times PC1+\left(\frac{Var\left(PC2\right)}{Var\left(PC1\right)+Var\left(PC2\right)}\right)\times PC2 \end{array}$$

Where Var(PC1) and Var(PC2) represent the variance explained by each component. This weighted approach ensures that the information from both primary components is adequately represented in the final score.

Finally, the PCA score was transformed to a scale of 0 to 100 to obtain the QCI, with higher values indicating better quality of care:


$$\begin{array}{l} QCI\left(x\right)=\frac{PCAscore\left(x\right)-min\left(PCAscore\right)}{max\left(PCAscore\right)-min\left(PCAscore\right)}\times 100 \end{array}$$

In this study, we calculated sex‐specific QCI across China, G20 and Global.

#### 2.2.6. Software

Trend analyses and factor decomposition were performed in R 4.4.2 (mgcv, joinpoint packages), and forecasting in Stata 18 MP. All statistical tests were 2‑sided with α = 0.05.

## 3. Results

### 3.1. Overall trends in MS burden (1990–2021)

From 1990 to 2021, the number of new MS cases in China increased from 1961 (95% UI: 1546–2436) to 2795 (95% UI: 2250–3347), representing a 42.5% increase. The age‑standardized incidence rate (ASIR) remained stable at 0.2 per 100,000 (EAPC = 0.4%, 95% CI: 0.4–0.5), significantly outpaced the global mean (0.1%). A 50.0% increase in new cases was observed globally over the same time period (41,970–62,920). The G20 countries accounted for 70% of the global cases but maintained an age-standardized incidence rate (ASIR) of 0.9/100,000 (EAPC = 0.0%). The disease burden in China has demonstrated a particularly pronounced increase: Case numbers rose from 17,887 (95% UI: 13,402–23,693) to 40,159 (95% UI: 31,807–50,038), while the age-standardized prevalence rate (ASPR) increased from 1.6 to 2.2 per 100,000 (EAPC = 1.8%, 95% CI: 1.7–1.9). This growth rate was twice the global average (EAPC = 0.9%), contrasting with the decline observed in G20 nations (ASPR decreased from 25.1 to 24.7 per 100,000; EAPC = −1.0%). Regarding health loss, China age-standardized disability-adjusted life years (DALYs) rate increased from 0.6 to 0.8 per 100,000 (EAPC = 1.7%). This trend diverged from global patterns where the rate declined from 12.8 to 11.4 per 100,000 (EAPC = −0.4%), and in G20 countries from 14.3 to 12.4 per 100,000 (EAPC = −0.6%). Mortality data revealed an increase in deaths in China from 47 to 110 cases. Although the absolute age-standardized mortality rate (ASMR) remained remarkably low (<0.05 per 100,000 throughout), regression analysis based on crude estimates indicated a statistically significant upward trend (EAPC = 2.0%). This increase may be primarily attributable to rising absolute mortality and demographic shifts. Globally, deaths increased from 9108 to 16,302 (ASMR = 0.2 per 100,000), with G20 countries accounting for 83.8% of deaths while maintaining a stable ASMR of 0.2 per 100,000 (Table 1)**.**

**Table 1:** All-ages cases, age-standardized incidence, prevalence, mortality, DALYs rate, and corresponding AAPC of multiple sclerosis in China, G20 countries and Global in 1990 and 2021.

Measures	Location	Number 1990	ASR 1990	Number 2021	ASR 2021	EAPC (95% CI)
		All-ages cases	Age-standardized rates	All-ages cases	Age-standardized rates	
		*n* (95% UI)				
Incidence	Global	41970.4 (36605.6–48234.5)	0.8 (0.7–0.9)	62920.1 (56015–70635.1)	0.8 (0.7–0.9)	0.1 (0.1–0.1)
	China	1961.3 (1546–2435.5)	0.2 (0.1–0.2)	2795.3 (2250.2–3346.7)	0.2 (0.1–0.2)	0.4 (0.4–0.5)
	G20	33200.1 (29130.1–37887.9)	0.9 (0.8–1)	43911.2 (39533.6–48621.2)	0.9 (0.8–1)	0.0 (0.0–0.1)
Prevalence	Global	1004659.7 (868374.4–1165224.7)	22.3 (19.3–25.6)	1887767.5 (1688654.3–2113707.8)	22.2 (19.8–24.8)	0.9 (0.8–0.9)
	China	17887.4 (13402–23693.1)	1.6 (1.2–2)	40159.4 (31806.9–50037.9)	2.2 (1.7–2.8)	1.8 (1.7–1.9)
	G20	845617.8 (731099.1–976298.5)	25.1 (21.8–28.8)	1472006.2 (1330972.9–1630956.7)	24.7 (22.2–27.4)	1.0 (0.9–1.0)
DALYs	Global	574234.3 (496155.6–662160)	12.8 (11.1–14.7)	973298.3 (838209.8–1133291.2)	11.4 (9.8–13.2)	0.4 (0.4–0.5)
	China	7015.6 (5078.1–9796.1)	0.6 (0.5–0.9)	15005.5 (11001.9–19590.3)	0.8 (0.6–1.1)	1.7 (1.6–1.9)
	G20	479420.9 (416814.8–551309.8)	14.3 (12.5–16.4)	759594 (650454.1–875738.9)	12.4 (10.6–14.4)	0.6 (0.5–0.7)
Deaths	Global	9107.8 (8711.1–9469.2)	0.2 (0.2–0.2)	16302.2 (15357.2–17039.5)	0.2 (0.2–0.2)	0.6 (0.5–0.7)
	China	47.4 (27.8–70.7)	0 (0–0)	109.8 (86–138.3)	0 (0–0)	2.0 (1.5–2.6)
	G20	7756.6 (7522–7991.2)	0.2 (0.2–0.3)	13660.3 (12769.3–14233)	0.2 (0.2–0.2)	0.9 (0.9–1.0)

95% UI = 95% uncertainty interval, AAPC = average annual percentage change, ASR = age‑standardized rate, DALYs = disability-adjusted life years, EAPC = average annual percentage change, G20 = group of 20.

### 3.2. Joinpoint regression analysis

Joinpoint regression revealed long-term declines in several indicators from 1990 to 2021, followed by reversals after 2021.

Incidence: Declines were observed globally (AAPC = −0.93%, *P = .039*), in the G20 (AAPC = −0.86%, 95% CI: −1.16 to − 0.74), and in China (AAPC = −0.50%, 95% CI: −0.65 to − 0.20). However, from 2019 to 2021, incidence increased (global APC = 6.23%, *P = .009*; G20 APC = 5.45%; China APC = 5.26%, *P = .004*).

Prevalence: Declines were observed globally (AAPC = −0.71%, *P* = .024) and in the G20 (AAPC = −1.19%, *P = .029*), while China showed a smaller decline (AAPC = −0.60%, *P = .028*). After 2019, prevalence increased globally (APC = 3.59%) and in the G20 (APC = 3.50%), whereas China decline moderated (APC = −0.41%).

DALYs: From 1990 to 2019, DALYs rates declined most rapidly in China (AAPC = −1.34%, 95% CI: −1.52 to − 1.07), followed by global (AAPC = −0.64%) and G20 (AAPC = −0.55%) trends. From 2019 to 2021, DALYs rates increased sharply in the G20 (8.84%, 95% CI: 8.46–9.24), China (5.14%, *P = .019*), and globally (+4.45%, *P = .004*).

**Figure 1.**
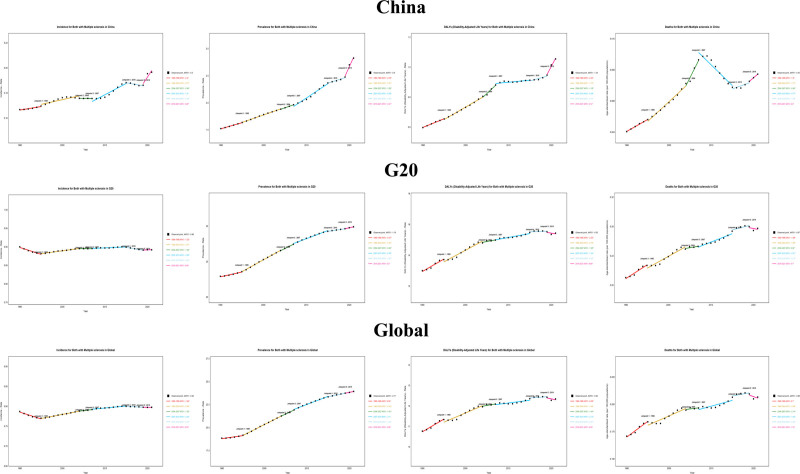
Joinpoint regression analysis of ASIR, ASPR, ASMR, and ASDR of multiple sclerosis in China, G20 countries and Global in 1990 and 2021. ASDR = disability‑adjusted life years rate, ASIR = age-standardized incidence rate, ASMR = age-standardized mortality rate, ASPR = age-standardized prevalence rate, G20 = group of 20.

Mortality: The largest mortality decline occurred in the G20 (AAPC = −0.67%, *P = .029*), followed by global (AAPC = −0.66%, 95% CI: −0.92 to − 0.38) and China (AAPC = −0.43%, 95% CI: −0.72 to − 0.19). After 2019, mortality rose most in the G20 (5.70%), followed by China (APC = 4.40%, *P = .027*) and globally (APC = 1.18%). See Figure 1**.**

### 3.3. Trend of disease burden

**Figure 2.**
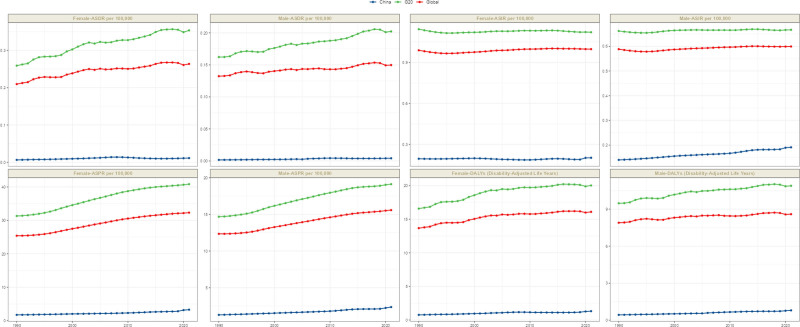
Trend of disease burden of ASIR, ASPR, ASMR, and ASDR of multiple sclerosis in China, G20 countries and Global in 1990 and 2021. ASDR = disability‑adjusted life years rate, ASIR = age-standardized incidence rate, ASMR = age-standardized mortality rate, ASPR = age-standardized prevalence rate, G20 = group of 20.

The global burden of MS has increased since 1990, but there is significant regional variation. China has experienced the fastest growth in MS-related health loss, with DALYs increasing by 84.95%/71.48% for both men and women, which far exceeds the global growth rate of 8.8%/17.73% and the growth rate in G20 countries of 14.96%/20.76%. Similarly, prevalence growth showed a gradient of China (>80% for both sexes) >G20 countries (~30%) >the global average (~27%), suggesting either prolonged survival or an improved diagnostic capacity for Chinese patients. There are gender differences in changes to morbidity: China has seen a 36.33% increase in males, compared to only 3.64% in females; globally, it has remained relatively stable (all <2%). Mortality rates were generally low in absolute terms (close to zero in China), but the rate of change was 157.19% in Chinese males, which may be related to a base effect. That is, due to the extremely low absolute number, a small increase can lead to a high percentage change. See Figure 2.

### 3.4. Age distribution of burden

The age-standardized disease burden analysis of MS from 1990 to 2021 revealed significant regional differentiation features: China showed a trend of younger disease spectrum. The peak age group shifted from 50 to 54 years in 1990 to 40 to 44 years in 2021. The 15 to 49 age group accounted for 68.3% of DALYs in 2021, up from 52.1% in 1990, with the largest increase in the 35 to 39 age group (148.7%).

**Figure 3.**
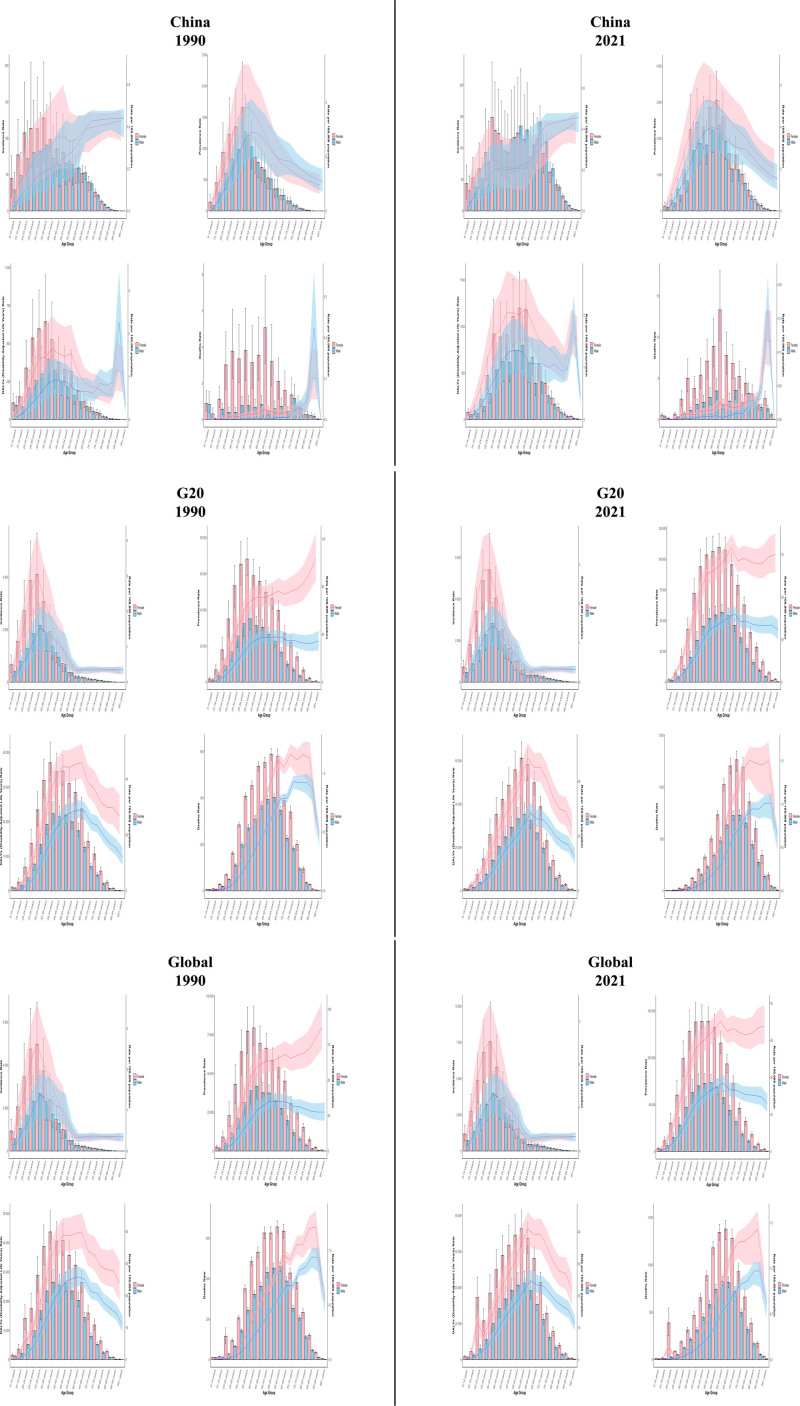
Age distribution of burden of incidence, prevalence, DALYs, and mortality of multiple sclerosis in China, G20 countries and global in 1990 and 2021. DALYs = disability-adjusted life years, G20 = group of 20.

In G20 countries, the peak remained at 45 to 49 years, but the ≥65 age group share rose from 12.7% to 24.1%. Globally, a bimodal pattern persisted (primary peak at 50–54 years; secondary at 25–29 years), with greater growth in the older peak. Regional comparisons reveal a concentrated burden among 25 to 54-year-olds in China (82.9% of the total), with a left-shifted curve pattern that differs significantly from the right-shifted expansion of the G20 (73.5% of the broad peak among 45–69-year-olds) and the global bimodal structure. In terms of gender, In China, women aged 30 to 44 had DALYs rates 1.8 to 2.3 times those of men, compared with 1.3 to 1.6 globally. The peak burden in Chinese women occurred 5 to 10 years earlier than in men. Further analysis of the data confirmed the polarization of China youthfulness’ and “aging in high SDI areas”: the proportion of DALYs in the 15 to 49 age group in China (68.3%) was 1.8 times higher than in the G20 countries, while the burden rate in the ≥ 70 age group in the G20 (12.4%) was 3.2 times higher than in China. See Figure 3.

### 3.5. Drivers of change

**Figure 4.**
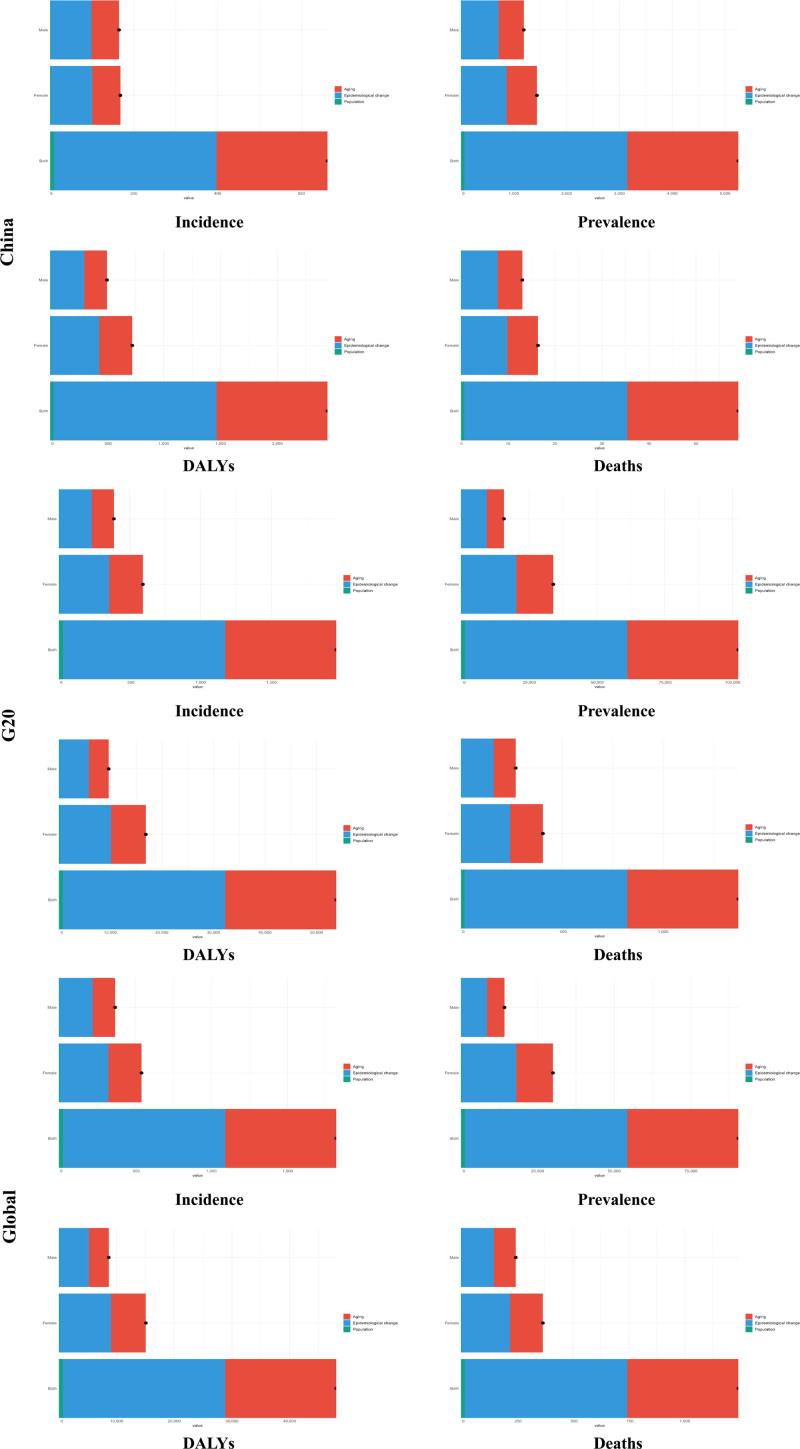
Drivers of change of incidence, prevalence, DALYs, and mortality of multiple sclerosis in China, G20 countries and global in 1990 and 2021. DALYs = disability-adjusted life years, G20 = group of 20.

From 1990 to 2021, the global burden of MS increased systematically, and the driving mechanisms showed common features across geographic strata. In China, the net changes in the 4 core indicators were as follows: incidence (165.11), prevalence (1312.32 per 100,000 people), DALYs (609.28) and mortality (14.79 per 100,000 people). The corresponding indicators in the G20 countries were 488.09; prevalence, 26,156.74/100,000; DALYs, 13,640.26; and mortality, 348.24/100,000. The global totals reached 454.19 for incidence; 23,144.68 per 100,000 for prevalence; 12,179.12 for DALYs; and 314.48 per 100,000 for mortality. Factor decomposition reveals that the 3 main drivers show cross-regional consistency. Epidemiological transitions (encompassing environmental risk exposure, diagnostic criteria refinement, healthcare accessibility improvements, and smoking disparities) constituted the dominant driver. In China, this factor accounted for 96.6% of incident case growth, while contributing 58.8% to 61.0% to increases in prevalence, DALYs, and mortality. Contributions remained stable at 58.5% to 58.7% globally and among G20 nations. Population aging emerged as the secondary driver (40.0%–66.0% contribution), while population growth exerted marginal effects (≤2.45%). Consistent gender disparities were observed across regions, with females experiencing a higher disease burden than males. Among Chinese females, net changes in all indicators exceeded those in males: Incidence (167.64 vs 164.54 per 100,000), prevalence(1438.43 vs 1188.56 per 100,000), DALYs (722.39 vs 500.14 per 100,000)and mortality (16.38 vs 13.05 per 100,000). This disparity was more pronounced in G20 nations, where female-to-male prevalence ratio reached 2.1 (33,973.62 vs 15,825.23 per 100,000). See Figure 4.

### 3.6. ARIMA projections (2022–2050)

**Figure 5.**
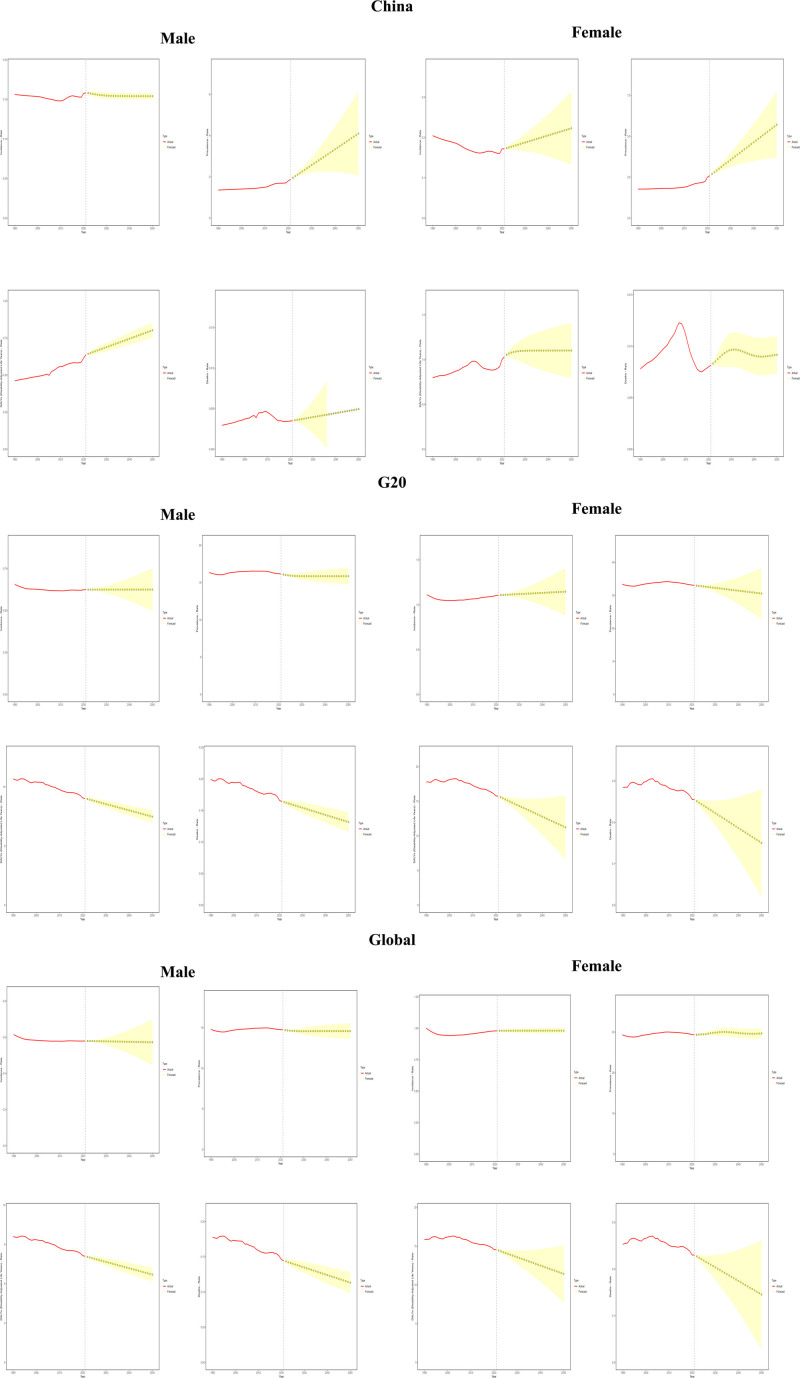
ARIMA projections (2022–2050) of incidence, prevalence, DALYs, and mortality of multiple sclerosis in China, G20 countries and global in 1990 and 2021. ARIMA = autoregressive integrated moving average, DALYs = disability-adjusted life years, G20 = group of 20.

This study systematically applied the ARIMA model to analyze cross-population projections of the global epidemiological burden of MS. The male incidence rate in China showed a slow decline (from 0.1579 in 2022 to 0.1540 per 100,000 in 2050; AIC = −342.02), whereas the female incidence rate increased (from 0.17–0.18 in 2022 to 0.13–0.31 per 100,000 in 2050; RMSE = 0.0025), reflecting a significant gender difference. The incidence rate for males in G20 countries remained stable (mean = 0.6246, MAPE = 0.087%), while the incidence rate for females in these countries increased moderately (from 1.108 in 2022 to 1.146 in 2050, MAPE = 9.31%). The global incidence rate for males declined slowly (from 0.579 in 2022 to 0.572 in 2050, MAPE = 6.3%), whereas the incidence rate for females showed a high degree of stabilization (mean annual increase of only 0.002%, ACF1 = 0.728). In terms of prevalence, there has been a significant increase in Chinese men (2.3% per annum, 2050 CI: 2.07–6.13) and an accelerated increase in women (3.4% per annum, 2050 CI: 3.69–7.72). There has been a slight decrease in G20 men (from 16.08 in 2022 to 15.86 in 2050) and a slow decrease in women (0.08 units per annum, The 2050 CI is 22.86–38.27. Global males show a decline followed by stabilization (stabilizing at 14.58–14.59 after 2026), while females show an increase followed by a decline (peaking at 30.01/100,000 in 2033). DALYs rates generally decline, with a significant rise in Chinese males (mean annual drift: 0.0057, *P* <*.05*), while rates for females rise slowly (to 1.10 in 2050, with a CI of 0.79–1.41). G20 men declined by 0.053 per annum (MAPE = 0.41%), and women declined even more dramatically (by 0.15 per annum). AIC = −36.57). Global males continued to decline by 4.8% p.a. with no upward inflection point, while females declined by 0.1 units p.a. Mortality trends diverged: a 0.9% increase for Chinese males (with a negative CI after 2037) and an inverted U-shaped curve for females (peaking at 0.0097 in 2031). There were significant declines for both the G20 and global populations (0.0011 per annum for global males). See Figure 5 and Table S1, Supplemental Digital Content, https://links.lww.com/MD/R177**.**

### 3.7. Age-QCI analysis (1990–2021)

**Figure 6.**
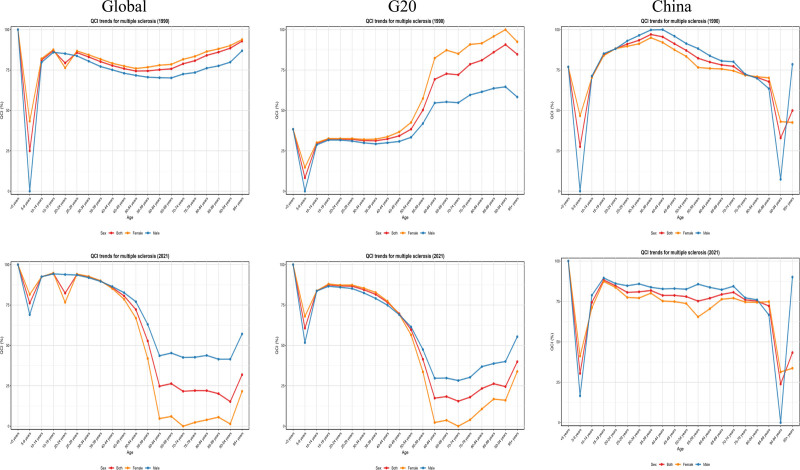
The trend of the QCI in China, G20 countries and global in 1990 and 2021. G20 = group of 20, QCI = quality of care index.

This study compared the QCI for MS in 1990 and 2021 across regions, age groups, and sexes. Globally, QCI values rose substantially (24.94–92.91 in 1990 to 76.18–94.60 in 2021), with adolescents showing the highest scores, while older adults remained disadvantaged. China demonstrated marked improvements, with all-age QCI increasing to 30.44 to 80.68 in 2021, particularly in the elderly (75–79 years, 72.08 to 75.99), and men consistently outperforming women (55–59 years, 85.70 vs 65.46). In G20 countries, QCI ranged from 8.31 to 87.50 in 2021, showing significant gains among younger children but persisting deficits in older groups. Overall, a “middle-high, both-ends-low” distribution was observed, with higher QCI in adolescents and middle-aged adults but lower values in young children and the elderly, and with men generally receiving better care than women. See Figure 6 and Table S2, Supplemental Digital Content, https://links.lww.com/MD/R177**.**

## 4. Discussion

Based on the GBD 2021 data, this study for the first time systematically revealed the temporal and spatial evolution patterns and driving mechanisms of the burden of MS in China, G20 countries, and globally from 1990 to 2021. The core findings show that the MS burden in China is characterized by rapid growth, structural heterogeneity, and obvious inflection points. In terms of absolute burden, the number of MS cases in China increased by 124.5%, the ASPR increased at an annual rate of 1.8%, which was twice the global average of 0.9%, and the rate of DALYs increased against the trend at an annual rate of 1.7%. This is significantly different to the global average of −0.4% and the average for G20 countries of −0.6%.

Joinpoint regression found that all 3 major regions showed a systematic reversal of disease burden indicators after 2019, particularly in terms of the rate of DALYs reversal. This was contrary to the predicted gradual decline in the global DALYs rate from the previous GBD study. Given the temporal coincidence with COVID-19 emergence, multiple pathways may explain this divergence: First, Pandemic-induced healthcare disruption potentially delayed MS diagnosis/treatment through chronic disease management collapse.^[37]^ Additionally, SARS‑CoV‑2 infection may exacerbate MS symptoms or trigger relapses.^[38,39]^ However, it remains uncertain whether this increased burden will persist, as pandemic-induced alterations in data collection systems and other factors may impact the accuracy and interpretation of subsequent results.

China MS disease burden is concentrated in the working‑age population (15–49 years), accounting for 68.3% of DALYs in 2021, with the peak age shifting from 50 to 54 years in 1990 to 40 to 44 years in 2021. In contrast, G20 countries demonstrated a progressive aging of the burden, with the proportion of DALYs in the ≥ 65 age group nearly doubling from 12.7% to 24.1%. This difference may reflect global disparities characterized by population aging in developed countries and disease rejuvenation in emerging economies.^[2,40,41]^

Complementing these burden dynamics, our QCI-based analysis highlights substantial improvements in care quality worldwide, with China showing particularly notable gains in elderly populations. Despite this progress, pronounced age- and sex-related disparities persist, as QCI values remain relatively low among children and older adults, and Chinese men consistently demonstrate higher QCI scores than women. Globally and in the G20, a “middle-high, both-ends-low” distribution was observed, indicating that adolescents and middle-aged adults benefit from superior care quality, whereas vulnerable groups such as the very young and the elderly continue to face care deficits. These findings suggest that, in addition to reducing disease burden, targeted interventions should address persistent inequities in care delivery, particularly for women and older populations.

Factor decomposition identified epidemiological changes as the primary driver of burden increases, followed by population aging, with females experiencing disproportionate escalation – Chinese women’s DALY increases exceeded males by 44.4%. Sex-specific decomposition further revealed that the contribution intensity of aging to the female burden consistently reached 1.5 to 2.1 times that observed in males across all regions: China, the G20, and globally. Gender disparities associated with epidemiological transition were even more pronounced. This consistent female-predominant gradient aligns with the established higher prevalence of MS among women. Potential underlying mechanisms involve sex hormone-mediated immunomodulation, differences in life expectancy, and heterogeneity in environmental exposures. These findings suggest that MS control strategies require integrated approaches addressing non-demographic drivers and incorporating sex-specific interventions.^[3,10,42,43]^

ARIMA modeling projections reveal significant geographical and sex-specific divergences in the global burden of MS. Regarding incidence, China exhibits divergent sex trajectories: a gradual decline in males contrasts with a fluctuating increase in females, potentially linked to fertility transitions and environmental estrogen exposures.^[42]^ Conversely, highly stable patterns were observed in G20 males and global females, suggesting epidemiological maturation in high-income regions.^[44,45]^ Prevalence growth shows a distinct gradient: significant increases occurred in China, aligning with population aging, whereas G20 and global rates plateaued or declined, indicating benefits of improved healthcare access. A notable regional paradox emerged in DALYs: Chinese males exhibited significant increases, contrasting sharply with global declines, highlighting critical rehabilitation resource shortages in LMICs.^[46]^ Mortality projections further validated this divergence: sustained increases in Chinese males versus significant global reductions, suggesting associations with socioeconomic factors or comorbid cognitive impairment in developing regions.^[47,48]^ Uncertainty analysis indicated widening long-term prediction intervals, primarily driven by 3 interactive factors: variations in disease natural history (gene-environment interactions), rates of diagnostic/therapeutic innovation, and the implementation intensity of social support policies.^[49,50]^ Our ARIMA projections should be interpreted as a baseline scenario that extrapolates historical patterns. They do not account for potential future disruptions or innovations. Nevertheless, these projections underscore the urgent need for sustained public health attention to MS in China.

Our study reveals a pronounced shift of MS burden toward younger populations in China, with 15 to 49-year-olds accounting for 68.3% of 2021 DALYs. This contrasts sharply with aging-driven epidemics in developed economies, where ≥ 65-year-olds represent 24.1% of G20 burden. Australia Greater Hobart cohort exemplifies this trend, showing a 206% prevalence surge and a quadrupled elderly proportion.^[51]^ Furthermore, the risk of MS increases significantly the further one lives from the equator, in both the southern and northern hemispheres, which is consistent with the latitudinal gradient effect in northern provinces of China (r_s_=0.93, *P* <.0001), aligning with global patterns.^[2,51–53]^ Critically, the post-2019 systemic reversal challenges classical MS progression models. European evidence links COVID-19-related care delays to doubled disability risk, suggesting public health emergencies exacerbate neurological damage through therapy discontinuation.^[54,55]^ Additionally, the cross-regional consistency of a significantly higher burden in women than in men is compatible with the sex hormone immunomodulation theory. However, the present study found that the peak burden in Chinese women of childbearing age occurs 5 to 10 years earlier than in men. This exceeds the scope of genetic predisposition explanations and reflects the synergistic effect of socio-behavior factors such as occupational stress and changes in reproductive patterns.^[24,56]^

This study achieves 3 key advancements beyond prior research: Methodologically, we innovatively integrate Joinpoint regression with ARIMA modeling to capture nonlinear MS burden dynamics, overcoming limitations of linear trend analyses in existing studies.^[57]^ Factor decomposition further quantifies 3 primary drivers (epidemiological transition/aging/population size), addressing Zhang et al omission of contribution metrics.^[2]^ Spatiotemporally, we pioneer the inclusion of COVID-19 pandemic data (2020–2021), revealing systemic healthcare disruption impacts – a gap in pre-2019 literature.^[2,57]^ Our novel G20 comparator framework transcends traditional “China versus global” dichotomies,^[2]^ identifying polarized burdens: aging-driven epidemics in developed nations versus youth concentration in China. At the applied value level, we establish a precision intervention framework targeting sex-age interactions. The prediction model showed that, in 2050, the prevalence rate of MS in women in China would be 1.6 times that in men, with peak burden occurring at 35 to 39 years – 10 years earlier than men. This provides empirical support for prioritizing access for reproductive-age women. Concurrently, we quantify the acute nerve damage impact of treatment interruption (diagnostic delays doubling disability risk), providing theoretical foundations for continuity-of-care protocols during public health emergencies.^[54]^

However, the study has several limitations. First, GBD estimates are influenced by the quality and availability of source data. In China, neurological specialty coverage is <30% in rural areas, which may lead to underdiagnosis, especially in younger age groups. Second, attributing the post‑2019 reversal solely to the COVID‑19 pandemic is difficult, as other factors – such as changes in reporting systems and healthcare policy – may have contributed.^[58,59]^ Third, the ARIMA model does not account for future policy changes or technological advances in diagnosis and treatment, which could alter projections.^[60,61]^ Fourth, while the GBD framework employs sophisticated modeling to address data gaps, the estimates for China may still be affected by underdiagnosis and misdiagnosis, particularly in rural areas where neurological specialty coverage is limited. This likely results in a conservative estimate of the true MS burden. Finally, registry coverage for MS in China remains limited, with <15% of provinces maintaining comprehensive patient registries. Additionally, the GBD 2021 dataset, which forms the basis of our analysis, does not provide subnational (e.g., provincial-level) or urban-rural disaggregated estimates for MS. Consequently, we were unable to explore how gradients in healthcare resource allocation, neurologist density, or diagnostic capacity might be driving the observed national trends.

Enhancing the national rare disease registry to integrate health insurance, electronic medical records, and real‑world research modules could improve surveillance and estimation accuracy. Prospective cohort studies are needed to quantify the effects of delayed diagnosis and treatment on disability progression. Furthermore, incorporating environmental and policy variables into projection models could support more robust scenario planning for MS prevention and control.

## 5. Conclusions

Our analysis demonstrates that the burden of MS in China has grown more rapidly than in G20 countries and globally, with a notable shift toward younger age groups and a greater impact on working‑age adults. In contrast, high‑income countries show a more pronounced aging of the MS burden. The systemic reversal in burden trends after 2019 is a notable finding. While its timing coincides with the COVID-19 pandemic, which may have contributed through disruptions in care, alternative explanations must be considered, including ongoing improvements in diagnostic awareness and healthcare access in China. The relative contribution of these factors remains to be elucidated in future studies. Epidemiological changes and population aging were the primary drivers of burden increases, with a disproportionately higher impact on females. While MS care quality rose from 1990 to 2021, with notable progress in China, stark global disparities remain. Women, the very young, and the elderly consistently receive lesser care. This pattern reveals a systemic issue where vulnerable populations are underserved compared to healthier adolescent and adult groups. These findings underscore the need for age‑ and sex‑specific prevention and management strategies, expanded access to disease‑modifying treatments, and improved healthcare infrastructure – particularly in neurology and rehabilitation services. Strengthening surveillance systems and integrating real‑world clinical data will be essential for monitoring trends and guiding targeted interventions to promote equity in MS care.

## Acknowledgments

The authors sincerely thank the GBD team for allowing us to access their comprehensive database.

## Author contributions

**Conceptualization:** Haosen Liao, Xiaomin Zhu.

**Data curation:** Haosen Liao, Xiaomin Zhu, Yuehui Ma.

**Formal analysis:** Haosen Liao, Cuilan Chen, Yingrui Huang, Honghu Wang, Hongen Yan.

**Funding acquisition:** Zheyi Zhou.

**Investigation:** Xiaomin Zhu, Cuilan Chen.

**Methodology:** Haosen Liao, Guanhua Li, Cuilan Chen.

**Project administration:** Zheyi Zhou.

**Software:** Haosen Liao, Xiaomin Zhu, Cuilan Chen, Yuehui Ma, Honghu Wang.

**Supervision:** Linglu Dun, Zheyi Zhou.

**Validation:** Hongen Yan.

**Visualization:** Yingrui Huang.

**Writing – original draft:** Haosen Liao, Xiaomin Zhu, Cuilan Chen.

**Writing – review & editing:** Zheyi Zhou.

## Supplementary Material


